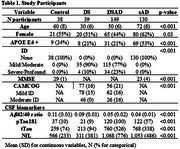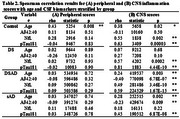# Fluid biomarkers of peripheral and central inflammation over the Alzheimer's disease continuum in adults with Down syndrome

**DOI:** 10.1002/alz70856_099361

**Published:** 2025-12-24

**Authors:** Olivia Belbin, Florencia Iulita, Laura Videla, Isabel Barroeta, Bessy Benejam, Miren Altuna, Lucía Maure‐Blesa, Íñigo Rodríguez‐Baz, Aida Sanjuan Hernandez, Laura Del Hoyo, Natalia Valle‐Tamayo, Oriol Dols‐Icardo, Alexandre Bejanin, Daniel Alcolea, Alberto Lleó, Juan Fortea, Maria Carmona‐Iragui

**Affiliations:** ^1^ Sant Pau Memory Unit, Hospital de la Santa Creu i Sant Pau, Institut de Recerca Sant Pau ‐ Universitat Autònoma de Barcelona, Barcelona, Spain; ^2^ CIBERNED, Network Center for Biomedical Research in Neurodegenerative Diseases, National Institute of Health Carlos III, Madrid, Spain; ^3^ Barcelona Down Medical Center, Fundació Catalana Síndrome de Down, Barcelona, Spain

## Abstract

**Background:**

Down syndrome (DS) is a genetic form of Alzheimer's disease (AD) and is associated with inflammation both dependent and independent of AD. This study aims to determine the contribution of DS and AD pathology to inflammation profiles in CSF and blood.

**Method:**

We applied the Olink Target 96 Inflammation panel to paired blood plasma and CSF from non‐trisomic cognitively unimpaired controls (*n* = 38), adults with DS with AD biomarkers within the normal range (DS; *n* = 39, all asymptomatic AD), DS with abnormal AD biomarkers (DSAD; *n* = 149, including asymptomatic and symptomatic AD), and patients with sporadic AD (sAD; *n* = 130) shown in Table 1. We used the first principal component of inflammation markers in plasma and CSF to generate peripheral and central inflammation scores, linear regression for group comparisons adjusting for covariates, Spearman test for correlation analyses and controlled the false discovery rate (FDR) using the Benjamini‐Hochberg method.

**Result:**

Trisomy 21 (DS versus controls) was associated with higher peripheral inflammation scores (*p* = 0.04), higher individual plasma markers (17 *p* <0.05, 3 FDR *p* <0.05), lower CNS inflammation scores (*p* = 0.01) and lower individual CSF markers (23 *p* <0.05, 10 FDR<0.05). Peripheral (rho=0.43) and CNS scores (rho=0.38) correlated with age in controls but not DS (rho<0.06). Compared to DS, DSAD showed higher peripheral scores (*p* = 0.04), higher individual plasma markers (14 *p* <0.05, 7 FDR *p* <0.05), higher CNS scores (*p* <0.0001) and higher individual CSF markers (37 *p* <0.05, 10 FDR *p* <0.05). sAD showed comparable peripheral scores (*p* = 0.16), individual plasma markers (30 *p* <0.05, 0 FDR>0.05) and CNS scores (*p* = 0.84), while individual CSF markers were elevated (39 *p* <0<05, 1 FDR>0.05) compared to controls. CNS but not peripheral scores correlated with age in DSAD (rho=0.24, rho=0.03) and sAD (rho=0.28, rho=0.03). CNS scores correlated with CSF pTau181 and NfL but not Aβ42:40 in DS and sAD and with all three biomarkers in DSAD (Table 2). Plasma scores did not correlate with CSF biomarkers in DS, DSAD or sAD (*p* >0.10).

**Conclusion:**

Inflammation was detectable in plasma and CSF in adults with DS prior to AD onset and over the AD continuum. Inflammation scores in the CSF reflect AD pathophysiological changes in DSAD even prior to AD onset.